# Associations between empirically derived dietary patterns and oxidative stress and inflammation in adults with primary hypothyroidism: a case-control study

**DOI:** 10.1186/s12902-023-01348-9

**Published:** 2023-05-10

**Authors:** Sorour Taherinia, Zahra Heidari, Rezvan Salehidoost, Mozhgan Karimifar, Arman Arab, Shadia Hamoud Alshahrani, Gholamreza Askari

**Affiliations:** 1 Isfahan Endocrine and Metabolism Research Center, Isfahan University of Medical Sciences, Isfahan, Iran; 2 Department of Community Nutrition, School of Nutrition and Food Sciences, Isfahan University of Medical Sciences, Isfahan, Iran; 3 Department of Biostatistics and Epidemiology, School of Health, Isfahan University of Medical Sciences, Isfahan, Iran; 4 Medical Surgical Nursing Department, King Khalid University, Khamis Mushate, Saudi Arabia

**Keywords:** Primary hypothyroidism, Dietary patterns, Stress oxidative, Inflammation

## Abstract

**Background:**

Primary hypothyroidism is a common endocrine disorder caused by impaired production of thyroid hormones. Recent studies have shown that dietary habits, oxidative stress, and inflammation may play roles in thyroid hypofunction. Thus, the present article aimed to determine the relationship between major dietary patterns and oxidative stress and inflammation in primary hypothyroid patients and healthy people in Iranian adults.

**Methods:**

This matched case-control study was conducted on 200 participants (100 cases and 100 controls). The presence of primary hypothyroidism was determined by endocrinologists based on American Thyroid Association (ATA) criteria. Dietary intake was assessed using a validated 168-item, semi-quantitative food frequency questionnaire (FFQ). The principal component analysis (PCA) method was used to derive major dietary patterns. Statistical analysis was performed using logistic regression analysis, and the findings were reported using odds ratios (ORs) with 95% CIs.

**Results:**

We identified 2 major dietary patterns (i.e., healthy and Western dietary patterns). After adjusting for confounding variables, participants in the highest tertile of the healthy eating pattern had lower odds of primary hypothyroidism. Also, there was a significant relationship between total antioxidant capacity (TAC) levels and thyroid hypofunction; however, no significant correlation was seen between the Western dietary pattern and malondialdehyde (MDA) and C-reactive protein (CRP) with hypothyroidism.

**Conclusions:**

There were statistically direct associations between healthy dietary patterns (loaded with vegetables, nuts and seeds, fruits, dried fruits, olives, garlic, black pepper, starchy vegetables, low-fat dairy, and legumes) and increased TAC levels with a decreased risk of thyroid hypofunction. However, Western dietary patterns and MDA and CRP levels did not associate with an underactive thyroid.

**Supplementary Information:**

The online version contains supplementary material available at 10.1186/s12902-023-01348-9.

## Introduction

Primary hypothyroidism is a prevalent endocrine disorder caused by impaired production of thyroid hormones [1]. The prevalence of adult hypothyroidism has been estimated at 1-2% in two-thirds of the world’s population [2]. In Iran, the prevalence rates of subclinical and overt hypothyroidism have been reported to be 5.5% and 2%, respectively [3, 4]. Thyroid disorders are the second most common metabolic disease after diabetes in the world [4, 5]. Women and the elderly are more affected by this disease [2]. Complications of untreated hypothyroidism include dyslipidemia, hypertension, cardiovascular disease, and mental disorders [6, 7].

Studies have shown that some hypothyroidism-related complications (such as endothelial dysfunction and cardiac atherosclerosis) are caused by inflammation and oxidative stress [8]. These complications (such as atherosclerosis) develop gradually, and inflammation plays an important role in all of its stages [9, 10]. Inflammation and oxidative stress can lead to hormonal disorders (such as hypothyroidism) and an imbalance between peroxidants and antioxidant defenses in the body [11].

Some studies have indicated that nutritional factors (such as low intake of fruits and vegetables) are associated with measured oxidative stress in the blood of patients with thyroid disorders [12, 13]. In recent years, dietary patterns have received much attention in examining the correlation between diet and health [14]. Since foods are not consumed alone and nutrients are metabolized together, it is necessary to evaluate the relationship between dietary patterns and the incidence of diseases than to evaluate each nutrient separately [15].

In a study, it was shown that a vegetarian diet, compared to an omnivorous diet, could reduce the risk of hypothyroidism, whereas the lacto-ovo diet was associated with an increased risk of hypothyroidism [16]. Another study on the relationship between dietary habits and oxidative stress in patients with Hashimoto’s thyroiditis (HT) suggested that low consumption of animal products had a protective effect, and dietary patterns could have a positive effect on the balance of the oxidative system in these patients [17].

Although investigating the relationship between dietary patterns and the etiology of diseases is of great importance, few studies have been conducted in this regard, especially in Iran. Accordingly, this study aimed to investigate the association between major dietary patterns and oxidative stress and inflammation in patients with primary hypothyroidism.

## Materials and methods

### Study design and population

In this case-control study, the relationship between major dietary patterns and antioxidant status and inflammatory factors in patients with primary hypothyroidism and healthy individuals has been investigated among Iranian adults. The case group included subjects whose thyroid stimulating hormone (TSH) levels were higher than the normal range (0.4-4 IU/L) [18]; they had been diagnosed less than a year with primary hypothyroidism based on American Thyroid Association (ATA) criteria [19]. The control group included subjects without primary hypothyroidism. Inclusion criteria were willingness to participate in the study and being at least 18 years old. Exclusion criteria were pregnancy, breastfeeding, using specific medications, following specific diets, and having chronic diseases, including cancer, diabetes, hyperthyroidism, goiter, thyroid nodules, and rheumatic, inflammatory, cardiovascular, renal, and liver diseases [15]. Further, subjects whose daily energy intakes were not in the range of 800-4,200 kcal/day and those who answered less than 35 food items on the food frequency questionnaire (FFQ) were also excluded from the study [20].

Using a simple random sampling method, a total of 200 participants (100 cases and 100 controls) were enrolled in this study. In the case group, 100 primary hypothyroidism patients were selected from the Isfahan Endocrine and Metabolism Research Center and Al-Zahra Hospital, both affiliated with Isfahan University of Medical Sciences. The disease was diagnosed by endocrinologists. In the control group, 100 subjects were selected from healthy relatives of patients referred to the center and Al-Zahra Hospital. Both groups were matched in terms of age and sex. Informed consent was obtained from all participants prior to their participation in this study according to the Helsinki Declaration. Moreover, STROBE (Strengthening the Reporting of Observational Studies in Epidemiology) guidelines were used to evaluate the quality of the paper [21]. The current study was approved by the Ethics Committee of Isfahan University of Medical Sciences (code: IR.MUI.RESEARCH.REC.1397.233).

### Dietary assessment

Dietary data were collected through individual interviews with participants using a semi-quantitative FFQ containing 168 food items. Previous studies have confirmed the validity and reliability of the questionnaire in the Iranian population [22]. The frequency of consumption of each food item of FFQ during the past year as daily, weekly, and monthly consumption was requested from the participants. The units used for each food item of FFQ were selected based on the criteria of nutritionists [23]. Then, using a modified nutritionist IV software (First Databank, Hearst Corp, San Bruno, CA, USA), the data obtained from the FFQ were converted to grams per day so that they could be compared with the same unit and determine the amount of total gram/energy, macronutrients, and micronutrients of each food item of the questionnaire [24].

### Biochemical assessment

TSH and anti-thyroid peroxidase (anti-TPO) antibody serum levels were determined using the medical records of participants. Their normal range was determined through the values defined for each commercial laboratory kit.

To measure the serum levels of the following biochemical data, 5 mL of venous blood was taken from the participants. Then, the samples were centrifuged for 10 min. Their sera were separated, transferred into 3 separate tubes, and stored at -80 °C.

Total antioxidant capacity (TAC) is a reliable indicator of the state of the antioxidant system [25]. Serum TAC was measured using a commercial assay kit (Kiazist Company, Hamedan, Iran). The basis of this method was the conversion of Cu^+ 2^ to Cu^+ 1^ by serum antioxidants using a chromogenic reagent that produces a colored complex at 450 nm; a standard Trolox curve was used to refer to the amount of serum TAC.

Malondialdehyde (MDA) is a significant indicator for assessing oxidative stress in the body, which is produced from lipid peroxidation [26]. Serum MDA was measured by a commercial laboratory kit (Kiazist Company, Hamedan, Iran). In this experiment, a colored complex measurable at 540 nm was formed by the reaction between MDA and thiobarbituric acid at 95 °C; a standard curve was used to measure serum MDA concentration.

C-reactive protein (CRP) is a sensitive and dependable protein indicator to measure the inflammatory status in the body [8]. Serum CRP was quantitatively measured by a commercial kit (Archem Kit, Asateb Company, Tehran, Iran). The immunoturbidimetric method was used for this assessment. Measuring the antigen-antibody reaction by the end-point method is the basis of this method.

### Assessment of other variables

The anthropometric characteristics of participants were measured using standard methods. Weight (Kg) was measured by a digital scale with an accuracy of 100 g with minimal clothing and no shoes. Height (cm) was measured by a tape measure without shoes in a standing position. Body mass index (BMI; kg/m^2^) was obtained by dividing weight (Kg) by height squared (m^2^) [27].

Demographic information of participants was recorded, including name, age, gender, marital status, and education. Also, the socioeconomic status of the participants was assessed through a questionnaire that included the type of residence, occupation, income, type of insurance, and whether they had a personal car and laptop or not. In addition, the medical and pharmacological history of the population was examined, such as type of disease, history of a specific drug, and supplement consumption. Participants were asked about smoking and alcohol use. In the end, blood pressure (**mm** Hg) was measured after 10 min of rest in a sitting position from the left arm using a sphygmomanometer.

### Statistical analysis

The sample size was estimated based on previous studies and a formula for comparison of the mean CRP index between case and control groups using α = 0.05, power = 0.8, SD = 4.8, and d = 1.9. The dropout rate of 10% was used, which yielded 220 subjects (110 subjects in each group) [28]. Moreover, 20 people (10 people from each group) were excluded from the study due to not accepting the blood test and not matching. Finally, 200 people (100 people in each group) participated in the current study. After selecting the case group, the matching process with the control group was done using statistical methods in terms of age and sex. In this way, the cases were classified with a gap of 5 years in terms of age, and the controls of the same age were matched with them; in addition, the number of women and men in both groups was selected equally. Exploratory factor analysis was used based on the principal component estimation method to identify major dietary patterns based on 27 food groups extracted from FFQ; this is because, among the main food groups (34 groups), those that had factor loadings of below 0.2 in the process of analysis were excluded to improve interpretability. First, the Kaiser-Meyer-Olkin criterion and Bartlett’s test of sphericity test were used to assess the quality of dietary data. The number of dietary patterns was obtained using a scree plot and eigenvalues in connection with the natural interpretation of factors. Eventually, the correlation between the factors was minimized and improved interpretability because factors were rotated using a varimax rotation. The extracted dietary patterns were named based on previous studies and food groups included in each pattern. The intake of food groups weighted by their factor loadings was summed to calculate the score of each dietary pattern. The study population was classified based on the tertiles of dietary pattern scores. The chi-square test was used to determine the distribution of participants across the 2 groups and the tertiles of dietary patterns according to categorical variables. The independent samples *t* test and 1-way analysis of variance (ANOVA) were used to assess the differences of continuous variables between the 2 groups and the categories of dietary patterns, respectively. The adjusted model controlled for age, gender, BMI, energy intake, systolic/diastolic blood pressure (SBP/DBP), history of the disease (yes/no), and smoking use (yes/no(. SPSS version 26 (SPSS Inc., Chicago, IL, USA) was used to analyze the data. *P* values less than 0.05 were considered statistically significant.

## Results

**Fig. 1:**
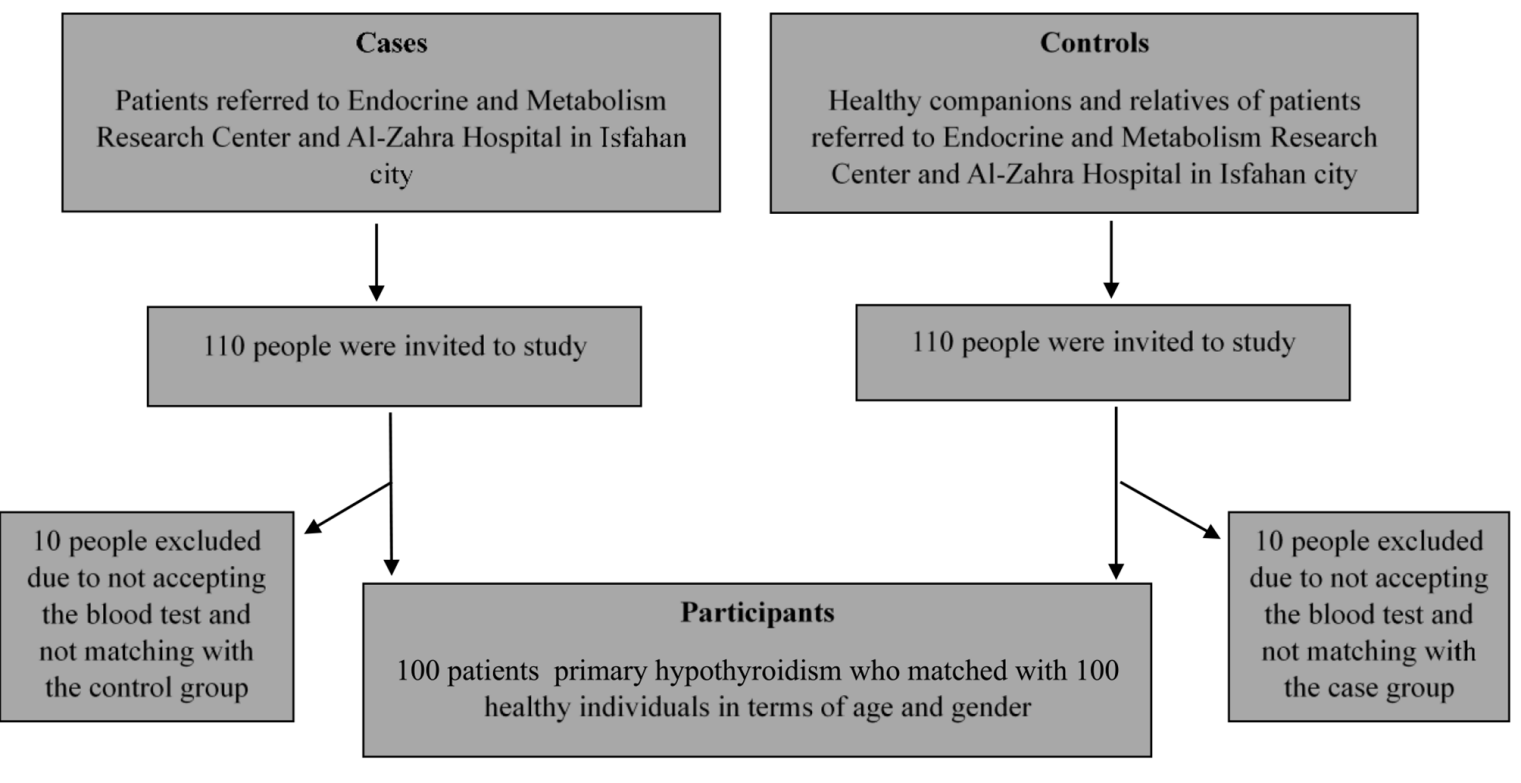
Flow diagram of the case-control study

Considering the possibility of dropouts, 220 people participated in this research, of whom 20 people were excluded from the study, and, finally, 200 people (100 cases and 100 controls) participated in this study (Fig. 1). The 2 major dietary patterns were identified using factor analysis among participants (Table 1). These dietary patterns were labeled as healthy and Western. A healthy dietary pattern was rich in organ meats, fish, low-fat dairy, fruits, dried fruits, vegetables, legumes, garlic, tomato, starchy vegetables, nuts, olives, pickles, broth, and black pepper. In Western dietary patterns, the intakes of processed meats, red meats, fish, high-fat dairy, fruit juices, compotes, fast food, salty snacks, mayonnaise, sugar-sweet dessert, solid fat, soft drinks, and salt were high. As presented in Table 1, each food item of the FFQ was allocated to one of 34 predefined food groups based on the similarity of the nutrients to identify major dietary patterns. According to our analysis, the total variance for healthy and Western dietary patterns was 12.34% and 11.79%, respectively.

**Table 1:** Food groups used for dietary pattern analysis and factor loadings for each of the identified dietary patterns

Food groups	Food items	Dietary patterns	
		Healthy	Western
Vegetables	Yellow, green leafy, and all other types of vegetables	0.71	
Nut and seeds	Walnuts, peanuts, pistachios, hazelnuts, almonds, seeds	0.63	
Fruits	Different kinds of fresh fruits	0.58	
Dried fruits	Raisin, dried berry, dried fig, dried peach and apricot sheet	0.53	
Tomato	Tomato, red Sauce	0.53	
Olive	Green olive, olive oil	0.49	
Garlic	Garlic	0.37	
Black pepper	Black pepper	0.36	
Pickles	All types of vegetable pickles	0.35	
Starchy vegetables	Potato, fava bean	0.32	
Low-fat dairy	Skimmed milk, low-fat milk and yogurt, cheese, dough,	0.32	
Organ meats	Liver, rumen rennet, tongue, brain, head, leg	0.31	
Broth	Broth	0.23	
Legume	Lentil, beans, pea, split pea, mung bean, soya	-0.30	
Salty snacks	Cracker, chips, puff		0.65
Sugar-sweet-dessert	Confectionary products, sugar, jam, honey, candy		0.62
Soft drinks	All types of soft drinks		0.60
Processed meats	Sausage, kielbasa		0.54
Mayonnaise	Mayonnaise Sauce		0.48
Compote	Fruit compote		0.47
Fast food	French fries, pizza, hamburger		0.43
Fish	All types of fish, canned tuna	0.40	0.43
Red meats	Beef veal, lamb meat, mince meat		0.42
Fruit juices	All kinds of natural juices		0.41
Solid fat	Butter, margarine, piyeh, animal oil, solid oil		0.35
Salt	Salt		0.30
High-fat dairy	High-fat milk and yogurt, cream cheese, cream, all kinds of flavored milk and yogurt, all kinds of ice cream		0.28
% of variance explained		12.34	11.79

*Factor loadings of < 0.2 have been removed to simplify the table

The comparison of energy and macronutrient intake, allergens, goitrogens, major food groups, and dietary patterns scores between case and control groups were illustrated in Table S1. The mean consumption of the refined grain group in the case group was significantly more than the control group. The mean consumption of fruit, dried fruit, and olive groups, as well as the healthy dietary pattern score, was significantly higher in the control group than in the case group (all *P* values < 0.05). There was no significant relationship between other variables in both groups (all *P* values ˃ 0.05). Furthermore, regarding allergens and goitrogens, no significant association was observed between the 2 groups (all *P* values > 0.05).

**Fig. 2:**
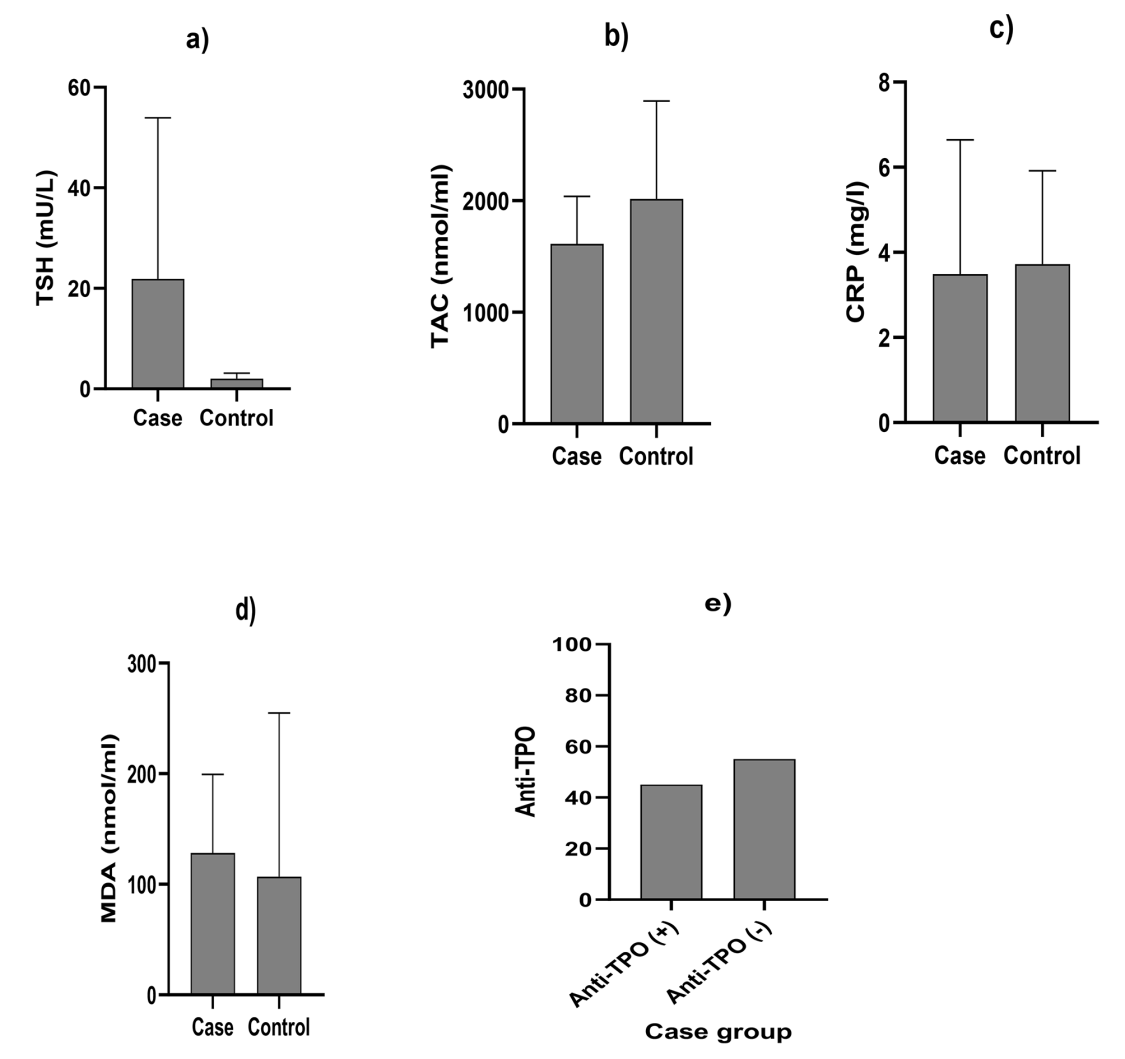
Comparison of biochemical variables in the case and control groups (Mean ± SD or Number). There were significant differences in TSH, TAC, and CRP between case and control groups (all P values < 0.05), but there was no significant difference in MDA between cases and controls (all P values > 0.05). 45% of people with hypothyroidism had anti-TPO positive (+) and 55% of them were with anti-TPO negative (-). (**a**). TSH, (**b**). TAC, (**c**). CRP, (**d**). MDA, (**e**). Anti-TPO

The comparison of biochemical variables between the case and control groups is indicated in Fig. 2. There were significant differences in TSH, TAC, and CRP between the case and control groups (all *P* values < 0.05), but there was no significant difference in MDA between the 2 groups (*P* value > 0.05). Furthermore, the distribution of anti-TPO antibodies among participants is presented in Fig. 2. This factor was evaluated to investigate the etiology of hypothyroid patients. The findings showed that 45% of people with primary hypothyroidism had anti-TPO positive (+); thus, these patients were suffering from HT, and 55% of them had anti-TPO negative (˗).

The socioeconomic status of the participants and general, demographic, and health-related characteristics are shown in Table 2. The socioeconomic status of the participants was obtained through the evaluation of variables such as house, job, income, laptop, car, and insurance. There were significant differences in height, disease history, drug use, and smoking between the case and control groups (all *P* values < 0.05). However, no significant difference was observed in other variables such as age, weight, BMI, blood pressure, gender, marital status, education, history of dieting, supplement use, alcohol, and socioeconomic status (all *P* values > 0.05).

**Table 2:** General characteristics of the study population

Variables	Case (n = 100)	Control (n = 100)	P value
Age (year)	42.12 ± 12.52	42.85 ± 12.4	0.68
Weight (kg)	70.75 ± 12.76	71.35 ± 13.55	0.75
Height (cm)	165.24 ± 9.01	163 ± 9.3	< 0.001
BMI (kg/m^2^)	25.92 ± 4.48	26.90 ± 4.75	0.135
SBP (mm/Hg)	120.91 ± 9.91	120.46 ± 10.15	0.75
DBP (mm/Hg)	80.33 ± 6.30	70.61 ± 0.93	0.258
**Gender**			
Female	75 (75%)	75 (75%)	
Male	25 (25%)	25 (25%)	>0.99
**Marital status**			
Single	14 (14%)	12 (12%)	0.341
Married	86 (86%)	86 (86%)	
Divorced	0 (0%)	2 (2%)	
**Education**			
Under diploma	39 (39%)	26 (26%)	0.094
Diploma	33 (33%)	34 (34%)	
Collegiate	28 (28%)	40 (40%)	
**History of disease**			
No	62 (62%)	83 (83%)	0.001
Yes	38 (38%)	17 (17%)	
**History of dieting**			
No	91 (91%)	84 (84%)	0.134
Yes	9 (9%)	16 (16%)	
**Drug use**			
No	57 (57%)	72 (72%)	0.027
Yes	43 (43%)	28 (28%)	
**Supplement use**			
No	60 (60%)	54 (54%)	0.391
Yes	40 (40%)	46 (46%)	
**Smoking**			
No	100 (100%)	94 (94%)	0.013
Yes	0 (0%)	6 (6%)	
**Alcohol**			
No	98 (98%)	99 (99%)	0.561
Yes	2 (2%)	1 (1%)	
High	33 (33%)	40 (40%)	0.532
Moderate	45 (45%)	38 (38%)	
Low	22 (22%)	22 (22%)	

Data are presented as mean ± standard error or number (%)

P-values are based on an independent sample t-test and the Chi-square test

BMI Body Mass Index, SBP Systolic Blood Pressure, DBP Diastolic Blood Pressure

P < 0.05 was considered statistically significant

General characteristics, biochemical variables, socioeconomic status, demographic characteristics, health-related characteristics, and macro- and micronutrient intake of the study population across tertiles of the dietary patterns are indicated in Table 3. Based on this table, there are significant associations in age, marital status, education, disease history, drug use, and smoking across tertiles of the Western dietary pattern in the participants (all *P* values < 0.05). However, no significant associations were seen in weight, height, BMI, blood pressure, gender, socioeconomic status, history of dieting, supplement use, and alcohol across tertiles of the dietary patterns in the participants (all *P* values > 0.05). Regarding macro- and micronutrient intake, there were significant differences in energy intake, carbohydrate, fat, saturated fatty acid (SFA), monounsaturated fatty acid (MUFA), polyunsaturated fatty acid (PUFA), zink, and vitamins D and C across the highest tertile and fiber among the lowest tertile of the healthy dietary pattern in the study population; moreover, subjects in the highest tertile of the Western dietary pattern had higher intake calories, protein, fat, cholesterol, SFA, zink, and vitamins A, D, and C compared to those in the lowest tertile (all *P* values < 0.05). No significant differences were seen in terms of other macro- and micronutrient intake across tertiles of dietary patterns (all *P* values > 0.05).

**Table 3:** Demographic and clinical characteristics of the study population across tertiles of the dietary patterns

Healthy dietary pattern					Western dietary pattern			
Variables	Tertile 1	Tertile 2	Tertile 3	P value	Tertile 1	Tertile 2	Tertile 3	P value
Age (year)	42.84 ± 11.88	41.23 ± 10.96	43.38 ± 14.31	0.588	50.06 ± 12.74	39.67 ± 10.70	37.29 ± 9.69	< 0.001
Weight (kg)	72.77 ± 14.91	69.09 ± 11.96	71.29 ± 12.24	0.265	70.91 ± 13.80	73.07 ± 11.11	69.20 ± 14.12	0.241
Height (cm)	165.61 ± 8.62	162.88 ± 8.76	163.85 ± 10.1	0.220	162.57 ± 8.69	165.15 ± 9.43	164.71 ± 9.42	0.217
BMI (kg/m^2^)	26.57 ± 5.19	26.02 ± 4.06	26.65 ± 4.62	0.699	26.87 ± 5.11	26.92 ± 4.53	25.43 ± 4.09	0.108
SBP (mm/Hg)	130.19 ± 12.34	110.6 ± 1.31	130.26 ± 12.17	0.556	110.98 ± 1.71	130.29 ± 12.26	120.81 ± 12.43	0.745
DBP (mm/Hg)	70.61 ± 0.1	70.64 ± 0.91	80.68 ± 7.71	0.301	70.78 ± 0.9	80.75 ± 7.78	70.41 ± 0.84	0.215
TAC (nmol/mL)	7.49 ± 0.41	7.39 ± 0.31	7.45 ± 0.28	0.235	7.46 ± 0.32	7.39 ± 0.37	7.47 ± 0.33	0.351
MDA (nmol/mL)	4.67 ± 0.56	4.68 ± 0.66	4.68 ± 0.64	0.988	4.77 ± 0.6	4.57 ± 0.62	4.68 ± 0.63	0.189
CRP (mg/L)	1.08 ± 0.77	0.98 ± 0.71	1.03 ± 0.71	0.713	0.91 ± 0.8	1.02 ± 0.69	1.17 ± 0.67	0.118
Socioeconomic status				0.186				
High Moderate Low	28 23 16	22 35 10	23 25 18		23 27 19	23 28 14	27 28 11	0.639
Gender				0.135				0.920
Female Male	45 22	55 12	50 16		39 12	40 12	36 12	
Marital status				0.268				0.034
Single Married Divorced	7 60 0	11 56 0	8 56 2		0 51 0	7 44 1	9 39 0	
Education								
Under diploma	25	22	18	0.271	27	15	14	0.003
Diploma Collegiate	26 16	19 26	22 26		14 10	22 15	16 18	
History of disease				0.473				
No Yes	51 16	45 22	49 17		29 22	40 12	35 13	0.02
History of being on a diet				0.106				
No Yes	63 4	55 12	57 9		44 7	45 7	42 6	0.95
Drug use				0.411				
No Yes	43 24	47 20	39 27		21 30	38 14	32 16	0.001
Supplement use				0.099				
No Yes	45 22	33 34	36 30		25 26	32 20	29 19	0.574
Smoking				0.129				
No Yes	63 4	67 0	64 2		51 0	51 1	48 0	0.007
Alcohol				0.364				
No Yes	65 2	67 0	65 1		50 1	52 0	48 0	0.283
Total energy (kcal/d)	2969.03 ± 756.40	3170.04 ± 708.77	3669 ± 550.07	< 0.001	3181.63 ± 739.79	3102.14 ± 732.65	3519.704 ± 676.83	0.002
Protein (g/d)	62.16 ± 20.74	66.43 ± 23.10	70.86 ± 21.88	0.075	60.35 ± 20.41	60.6 ± 18.82	78.63 ± 22.08	< 0.001
Carbohydrate (g/d)	335.46 ± 97.26	361.97 ± 102.26	419.14 ± 92.16	< 0.001	368.52 ± 108.04	354.92 ± 105.29	392.32 ± 92.75	0.108
Fat (g/d)	145.25 ± 48.99	162.35 ± 47.97	194.61 ± 50.18	< 0.001	162.88 ± 52.57	157.32 ± 53.85	181.65 ± 50	0.021
Cholesterol (mg/d)	278.93 ± 282.05	297.34 ± 231.23	284.01 ± 241.64	0.91	159.95 ± 130.53	256.04 ± 230	449.63 ± 280.69	< 0.001
Saturated fatty acid (g/d)	30.97 ± 12.04	34.26 ± 10.14	39.75 ± 10.33	< 0.001	32.92 ± 9.96	32.59 ± 10.23	39.45 ± 12.69	< 0.001
Mono-unsaturated fatty acid (g/d)	55.20 ± 20.26	62.04 ± 19.39	74.56 ± 22.04	< 0.001	63.37 ± 21.9	59.89 ± 21.86	68.36 ± 21.74	0.085
Polyunsaturated fatty acid (g/d)	42.82 ± 17.11	47.95 ± 16.43	58.93 ± 19.26	< 0.001	50.17 ± 19.29	47.08 ± 19.19	52.25 ± 17.75	0.287
Vitamin A (RAE) (µg/d)	1199.45 ± 1769.22	1157.61 ± 1066.38	1077.16 ± 581.85	0.847	868.09 ± 625.67	970.1 ± 739.82	1606.98 ± 1844.52	0.001
Vitamin D (µg/d)	0.1 ± 0.12	0.17 ± 0.2	0.18 ± 0.18	0.009	0.1 ± 0.13	0.1 ± 0.1	0.25 ± 0.23	< 0.001
Vitamin C (mg/d)	53.25 ± 25.76	76.78 ± 30.71	103.91 ± 36.13	< 0.001	70.82 ± 39	70.94 ± 31.92	92.01 ± 36.78	0.001
Zink (mg/d)	9.72 ± 3.89	10.66 ± 3.56	11.32 ± 3.52	0.043	9.74 ± 3.39	9.6 ± 3.41	12.37 ± 3.68	< 0.001
Iron (mg/d)	19.56 ± 12.85	18.66 ± 6.25	21.89 ± 16.24	0.307	20.18 ± 16.15	17.8 ± 6.21	22.06 ± 12.52	0.148
Selenium (µg/d)	114.64 ± 55.27	106.58 ± 50.47	103.1 ± 50.5	0.425	106.62 ± 50.43	100.94 ± 55.61	116.8 ± 49.74	0.211
Total fiber (g/d)	45.79 ± 31.08	36.79 ± 12.88	42.84 ± 12.33	0.04	39.53 ± 17.34	42.97 ± 28.59	43.03 ± 14.72	0.540

Data are presented as mean ± standard error or number (%)

P-values are based on ANOVA or chi-square test

BMI Body Mass Index, SBP Systolic Blood Pressure, DBP Diastolic Blood Pressure, TSH Thyroid Stimulating Hormone, TAC Total Antioxidant Capacity, MDA

Malondialdehyde, CRP C-Reactive Protein, RAE Retinol Activity Equivalents

P < 0.05 was considered statistically significant

The multivariable-adjusted odds ratios (ORs) for primary hypothyroidism according to tertiles of dietary patterns are illustrated in Table 4. In the crude and models 1 and 2, the lowest tertiles of adherence to dietary patterns (tertile 1) were defined as the reference. In the crude, participants in the highest tertile of healthy dietary patterns tended to have lower odds of primary hypothyroidism (OR = 0.44; 95% CI: 0.22; 0.88; *P*_trend_ = 0.02). In model 1, after controlling for age, gender, BMI, and energy intake, those in the highest tertile of the healthy dietary pattern had lower odds of primary hypothyroidism (OR = 0.45; 95% CI: 0.21; 0.96; *P*_trend_ = 0.044). Also, in model 2, after controlling for age, gender, BMI, energy intake, blood pressure (SBP/DBP), disease history, and smoking use, those in the highest tertile of the healthy dietary pattern had lower odds of primary hypothyroidism (OR = 0.38; 95% CI: 0.16; 0.88; *P*_trend_ = 0.026). In addition, there was no significant association between the Western dietary pattern score and hypothyroidism in crude and all models.

**Table 4:** Odds ratio and 95% confidence interval for primary hypothyroidism according to tertiles of major dietary patterns

Healthy dietary pattern					Western dietary pattern			
	**Tertile 1**	**Tertile 2**	**Tertile 3**	**P trend**	**Tertile 1**	**Tertile 2**	**Tertile 3**	**P trend**
Crude	Ref	1 (0.51, 1.98)	**0.44 (0.22, 0.88)**	**0.02**	Ref	1.54 (0.78, 3.04)	0.86 (0.43, 1.68)	0.668
Model 1	Ref	1.02 (0.50, 2.05)	**0.45 (0.21, 0.96)**	**0.044**	Ref	1.66 (0.78, 3.53)	0.87 (0.40, 1.87)	0.664
Model 2	Ref	0.81 (0.38, 1.74)	**0.38 (0.16, 0.88)**	**0.026**	Ref	2.03 (0.90, 4.60)	1.13 (0.49, 2.58)	0.815

Data are presented as odds ratio (95% confidence interval)

Crude: Unadjusted

Model 1: Controlled for age, gender, BMI, and energy intake

Model 2: Controlled for age, gender, BMI, energy intake, blood pressure (SBP, DBP), history of disease, and smoking

P < 0.05 was considered statistically significant

The multivariable-adjusted ORs for the association between antioxidant and inflammatory status variables with primary hypothyroidism are presented in Table 5. Based on the findings, increasing TAC levels reduces the odds of primary hypothyroidism. This relationship remains the same after adjusting for demographic variables, disease history, and smoking use. No significant relationship was observed between the MDA and CRP with thyroid hypofunction.

**Table 5:** Odds ratio and 95% confidence interval for the association between primary hypothyroidism and antioxidant and inflammatory status

	Crude	Model 1	Model 2
TAC (nmol/mL)	**0.99 (0.98, 0.99)**	**0.99 (0.98, 0.99)**	**0.98 (0.98, 0.99)**
MDA (nmol/mL)	1.02 (0.99, 1.05)	1.04 (1.01, 1.08)	1.05 (1.02, 1.10)
CRP (mg/L)	0.98 (0.88, 1.09)	0.98 (0.87, 1.09)	1.01 (0.90, 1.14)

Data are presented as odds ratio (95% confidence interval)

Crude: Unadjusted

Model 1: Controlled for age, gender, BMI, and energy intake

Model 2: Controlled for age, gender, BMI, energy intake, blood pressure (SBP, DBP), history of disease, and smoking

P < 0.05 was considered statistically significant

## Discussion

In the current case-control study, we extracted 2 dominant dietary patterns, i.e., healthy and Western dietary patterns. Our analysis demonstrated that adherence to a healthy eating pattern reduces the chance of primary hypothyroidism. However, there was no significant association between Western dietary patterns and primary hypothyroidism. The risk of primary hypothyroidism decreases by increasing TAC levels, though no significant relationship was seen between MDA and CRP with an underactive thyroid. The relationship between major dietary patterns and oxidative and inflammatory status in patients with thyroid hypofunction was discussed in this study for the first time in Middle Eastern countries, especially Iran.

Ruggeri et al. reported that low consumption of animal foods reduces thyroid autoimmunity and oxidative stress in this disease, while a plant-based diet had positive effects in these patients [17]. In addition, the number of final glycation products was significantly higher in HT than in healthy subjects, but the antioxidant activities of total plasma, thioredoxin reductase, and glutathione peroxidase were lower in the case group than in the control group, indicating oxidative stress [17]. Another study reported that the prevalence and incidence of hypothyroid disease were lower in vegetarians than in omnivores, even after adjusting for confounding factors [16]. Kalicanin et al. documented that there were positive associations between nuts, processed meats, and animal fats in HT patients than in healthy people; however, significant negative relationships were seen between the consumption of whole grains, fruits, vegetable oils, olive oil, red meats, oily fish, soft drinks, and liquor in the case group than in the control group [29]. Another dataset showed that oxidative stress was enhanced in women with HT after pharmacotherapy with levothyroxine, though the daily consumption of vegetables and fruits and maintaining weight within the normal range were effective in reducing their oxidative stress [12]. Another paper represented that euthyroid HT was associated with elevated oxidant and reduced antioxidant levels, indicating an oxidation state [30].

Our study showed a significant positive relationship between the healthy dietary pattern and TAC with primary hypothyroidism, which is in line with previous studies [12, 17, 30]. However, we did not find any significant association between the Western dietary pattern and MDA and CRP levels with primary hypothyroidism, which contradicts previous studies [29, 31, 32]. These discrepancies are due to differences in kits used to measure biochemical variables, covariates, food items within major dietary patterns, study design, statistical methods, study population, and nutritional assessment tools.

Studies on the role of major dietary patterns in oxidative stress and inflammation caused by hypothyroidism and its pathogenesis have conflicting results, but some possible mechanisms are possible for them. Fruits and vegetables, as part of healthy dietary patterns, are rich in fiber, carotene, folate, various phytochemicals/polyphenols, and vitamins B, C, and E [33, 34]. They play antioxidative, anti-inflammatory, and immunomodulatory roles in the body [35, 36]. Also, the beneficial effects of dried fruits result from gallic acid that exists in red fruits, apple peels, and grapes [36]. Gallic acid decreases the expression of the interleukin 6 (IL-6) gene, inhibiting the differentiation of T helper 17 (Th17) cells that intervene in the pathogenesis of autoimmune thyroid diseases (AITDs) [34, 37]. Western dietary patterns are rich in SFAs of animal origin (which negatively affect AITD), but anti-inflammatory diets (such as vegan diets) play useful roles in the pathogenesis of autoimmune disorders, including multiple sclerosis, rheumatoid arthritis, and systemic lupus erythematosus [34, 38–41].

Our findings also showed relationships between antioxidant and inflammatory factors with thyroid hypofunction. The imbalance between the production of free radicals and the function of the antioxidant system is defined as oxidative stress, whose role is discussed in the pathogenesis of some inflammatory and immune diseases such as AITDs [30, 42–47]. Environmental factors result in the excessive production of reactive oxygen species (ROS), leading to the modification of tissue proteins, the irregularity of the immune system, and the onset of autoimmune diseases (ADs); in addition, the aggravation of the pro-inflammatory state, tissue destruction, and the progression of ADs are other consequences of excessive production of ROS [48]. CRP is produced as an acute phase reactant in the liver and plays a role in response to inflammatory cytokines, including IL-6 [49]. Studies have shown that inflammatory cytokines are indicators of the risk of cardiovascular diseases; in addition, they increase in patients with HT [8, 49]. Enhanced CRP serum levels in HT patients are probably due to the impact of IL-6 on tumor necrosis factor α (TNF-α) and IL-1, as well as the reduction of the CRP clearance rate due to the decreased metabolic rate in these patients [50, 51].

The current study has some advantages. For instance, for the first time in the Middle East, this study was intended to evaluate the relationship between dietary patterns and oxidative and inflammatory indicators with primary hypothyroidism; in addition, many confounding variables were adjusted in the statistical analysis. However, this study has several limitations. First, the sample size of this study is small. Second, the assessment of dietary patterns by FFQ may elevate the risk of recall bias and under-over report of food items due to reporting dietary data during the past year. Third, limitations of using N4 software, which does not report some nutrients, such as iodine and trans fatty acids. Fourth, ignoring unknown confounders (such as mental status and genetic agents) may affect the results. Fifth, the impossibility of establishing a causal relationship between dietary patterns and the risk of hypothyroidism in case-control studies due to the inherent methodological limitations of these studies. Sixth, all cases were selected from one endocrine research center and one hospital in a city, and their healthy relatives were selected as controls; thus, generalizing the results to other populations should be done cautiously. Finally, anti-TPO serum levels were not measured in the control group, which may affect the findings.

## Conclusion

Adherence to a healthy dietary pattern rich in vegetables, nuts, seeds, fruits, dried fruits, olive, garlic, black pepper, starchy vegetables, low-fat dairy, and legumes reduces the chance of primary hypothyroidism. However, no correlation was observed between the Western dietary pattern and primary hypothyroidism among Iranians. Moreover, increased levels of TAC were associated with a reduced risk of thyroid hypofunction; however, there was no significant relationship between MDA and CRP with an underactive thyroid. Further studies with a longitudinal design are needed to confirm the current findings.

## Electronic supplementary material

Below is the link to the electronic supplementary material.

Supplementary Material 1

## Data Availability

Analyzed data relevant to the study are included in the article. The datasets generated are not publicly available as set out in agreements with the commercial partners but are available from the corresponding author on reasonable request.

## Abbreviations

FFQ: Food Frequency Questionnaire
PCA: Principal Component Analysis
OR: Odds Ratio
CI: Confidence Interval
TAC: Total Antioxidant Capacity
MDA: Malondialdehyde
CRP: C-Reactive Protein
HT: Hashimoto’s Thyroiditis
TSH: Thyroid Stimulating Hormone
IEMRC: Isfahan Endocrine and Metabolism Research Center
anti-TPO: Anti Thyroid Peroxidase
BMI: Body Mass Index
SBP: Systolic Blood Pressure
DBP: Diastolic Blood Pressure
SFA: Saturated Fatty Acid
MUFA: Mono-Unsaturated Fatty Acid
PUFA: PolyUnsaturated Fatty Acid
GFD: Gluten-Free Diet
AITD: Autoimmune Thyroid Disease
CD: Celiac Disease
fT4: Free Thyroxine
SCH: Subclinical Hypothyroidism
AIP: Autoimmune Protocol
anti-TG: Anti Thyroglobulin
CAT: Catalase
SOD: Superoxide Dismutase
IL-6: Interleukin-6
Th-17: T helper-17
ROS: Reactive Oxygen Species
ADs: Autoimmune Diseases
TNF-α: Tumor Necrosis Factor-Alpha
