# Supplementary material for: Associations between empirically derived dietary patterns and oxidative stress and inflammation in adults with primary hypothyroidism: a case-control study
Source: BMC Endocr Disord. 2023 May 10;23:105. doi: 10.1186/s12902-023-01348-9 (PMC10170704; doi:10.1186/s12902-023-01348-9)
Supplement: Supplementary file 1 — Supplementary Material 1 [file 12902_2023_1348_MOESM1_ESM.docx]

**Supplementary materials**


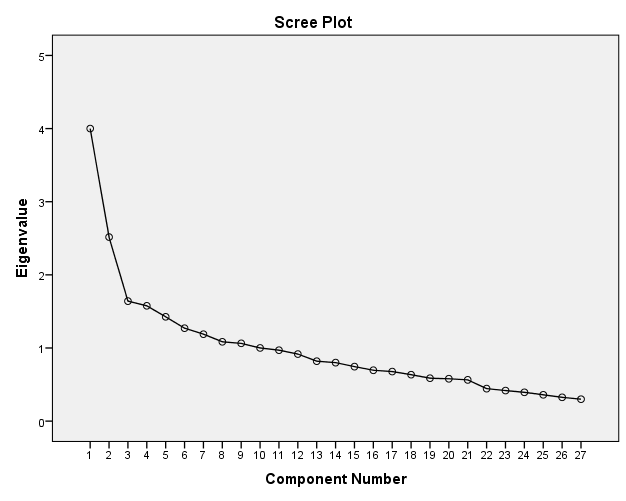


**Figure S1. The scree plot of the principal component analysis.**

**Table S1. Comparison of mean energy and macronutrient intake, allergens, goitrogens, major food groups, and dietary patterns scores in primary hypothyroid patients and healthy individuals**

| Variables | Case (n=100) | Control (n=100) | P value |
| --- | --- | --- | --- |
| Energy and macronutrient intake | | | |
| Protein (gr) | 64.30 ± 22.56 | 68.63 ± 21.53 | 0.167 |
| Fat (total) (gr) | 162.30 ± 51.20 | 172.24 ± 54.39 | 0.185 |
| Energy intake (kcal) | 3205.16 ± 691.28 | 3329.57 ± 776.86 | 0.233 |
| Carbohydrate (gr) | 367.46 ± 100.53 | 376.44 ± 105.64 | 0.539 |
| Allergens | | | |
| Wheat Flour | 17.77 ± 56.78 | 6.31 ± 15.26 | 0.056 |
| Peanut | 0.15 ± 0.4 | 0.4 ± 1.41 | 0.094 |
| Low-Fat Milk | 48.15 ± 92.55 | 70.36 ± 110.23 | 0.127 |
| Pistachios | 0.12 ± 0.45 | 0.27 ± 0.87 | 0.131 |
| Tuna | 1.61 ± 3.44 | 2.49 ± 5.15 | 0.159 |
| High-Fat Milk | 13.88 ± 32.16 | 21.09 ± 46.8 | 0.209 |
| Fish | 15.56 ± 23.84 | 20.15 ± 32.1 | 0.256 |
| Walnut | 2.21 ± 2.76 | 1.88 ± 2.51 | 0.37 |
| Soya | 17.23 ± 21.8 | 14.79 ± 20.15 | 0.419 |
| Hazelnut | 0.04 ± 0.2 | 0.06 ± 0.18 | 0.458 |
| Almond | 0.64 ± 1.87 | 0.84 ± 2.09 | 0.48 |
| Sausage | 1.35 ± 4.3 | 1.7 ± 4.34 | 0.575 |
| Egg | 16.43 ± 21.26 | 18.31 ± 34.29 | 0.644 |
| Kielbasa | 0.97 ± 5.93 | 0.71 ± 1.59 | 0.684 |
| Pizza | 18.62 ± 43.79 | 17.20 ± 21.5 | 0.773 |
| Beans | 30.08 ± 25.03 | 29.28 ± 26.37 | 0.827 |
| Hamburger | 1.30 ± 4.03 | 1.23 ± 2.02 | 0.871 |
| Corn | 1.60 ± 5.20 | 1.63 ± 4.28 | 0.968 |
| Goitrogens | | | |
| Turnip | 1.77 ± 5.25 | 3.29 ± 8.5 | 0.131 |
| Cabbage | 5.74 ± 12.72 | 8.34 ± 15.79 | 0.205 |
| Tea | 611.40 ± 531.51 | 523.04 ± 472.66 | 0.221 |
| Raw Spinach | 0.25 ± 1.78 | 0.45 ± 2 | 0.45 |
| Garlic | 0.73 ± 1.34 | 0.84 ± 1.47 | 0.583 |
| Coffee | 19.45 ± 79.35 | 24.43 ± 50.39 | 0.601 |
| Raw Onions | 7.84 ± 11.12 | 7.24 ± 9.62 | 0.688 |
| Major food groups | | | |
| Fruit group | **203.74 ± 98.74** | **252.17 ± 95.83** | **0.001** |
| Refined grain group | **270.75 ± 147.62** | **221.03 ± 114.65** | **0.009** |
| Olive group | **1.81 ± 3.38** | **3.07 ± 4.31** | **0.023** |
| Dried fruit group | **2.17 ± 3.61** | **3.51 ± 5.72** | **0.049** |
| Whole grain group | 128.11 ± 149.1 | 163.42 ± 131.14 | 0.08 |
| Soft drink group | 15.37 ± 30.47 | 24.55 ± 56.83 | 0.156 |
| Low-fat dairy group | 170.3 ± 162.43 | 202.36 ± 156.85 | 0.161 |
| Vegetable group | 163.27 ± 105.48 | 183.07 ± 109.33 | 0.196 |
| Fish group | 17.17 ± 26.98 | 23.08 ± 37.62 | 0.204 |
| Red meat group | 12.53 ± 9.12 | 15.02 ± 17.84 | 0.217 |
| High-fat dairy group | 57.7 ± 71.65 | 71.58 ± 93.73 | 0.244 |
| Salty snack group | 6.17 ± 12.72 | 4.66 ± 9.71 | 0.35 |
| Broth group | 16.51 ± 16.07 | 14.65 ± 14.30 | 0.388 |
| Solid fat group | 8.36 ± 13.08 | 7.11 ± 10.25 | 0.458 |
| Legume group | 163.83 ± 105.37 | 153.69 ± 132.95 | 0.552 |
| Suger, sweet, dessert group | 38.51 ± 39.43 | 35.71 ± 35.84 | 0.605 |
| Vegetable oil group | 5.78 ± 4.23 | 6.12 ± 5.6 | 0.631 |
| Garlic group | 0.73 ± 1.34 | 0.82 ± 1.45 | 0.654 |
| Egg group | 16.43 ± 21.26 | 18.14 ± 33.78 | 0.669 |
| Poultry group | 35.92 ± 33.32 | 33.49 ± 53.43 | 0.701 |
| Nut group | 6.77 ± 8.69 | 7.14 ± 7.06 | 0.745 |
| Fruit juices group | 9.63 ± 17.17 | 10.35 ± 14.08 | 0.746 |
| Tomato group | 86.29 ± 71.93 | 89.45 ± 106.81 | 0.807 |
| Organ meat group | 1.77 ± 3.48 | 1.85 ± 3.28 | 0.869 |
| Fast food group | 22.6 ± 45.15 | 22.94 ± 27.94 | 0.948 |
| Processed meats group | 2.32 ± 9.29 | 2.37 ± 5.35 | 0.964 |
| Starchy vegetables group | 19.56 ± 19.16 | 19.65 ± 20.95 | 0.977 |
| Dietary patterns scores | | | |
| Healthy dietary pattern score | **-0.14 ± 1** | **0.15 ± 0.98** | **0.039** |
| Western dietary pattern score | -0.04 ± 1.08 | 0.05 ± 1.08 | 0.522 |

Data are presented as mean ± standard error

P-values are based on an Independent sample t-test

P < 0.05 was considered statistically significant
